# Human Papillomavirus Vaccination Acceleration and Introduction in Sub-Saharan Africa: A Multi-Country Cohort Analysis

**DOI:** 10.3390/vaccines12050489

**Published:** 2024-05-01

**Authors:** Gbadebo Collins Adeyanju, Tene-Alima Essoh, Annick Raissa Sidibe, Furaha Kyesi, Muyi Aina

**Affiliations:** 1 Center for Empirical Research in Economics and Behavioural Science (CEREB), University of Erfurt, 99089 Erfurt, Germany; 2 Psychology and Infectious Disease Lab (PIDI), University of Erfurt, 99089 Erfurt, Germany; 3 Media and Communication Science, University of Erfurt, 99089 Erfurt, Germany; 4 Agence de Médecine Préventive (AMP) Afrique, Abidjan 08 BP 660, Côte d’Ivoire; tae@aamp.org; 5 National Immunization Technical Advisory Groups (NITAGs), Ouaga 06, Ouagadougou 06 BP 9096, Burkina Faso; annickraissa_s@yahoo.fr; 6 Ministry of Health, S.L.P. 743, Dar es Salaam P.O. Box 9083, Tanzania; furahakyesi@hotmail.com; 7 Executive Secretary, National Primary Healthcare Development Agency (NPHCDA), Area 11, Abuja P.O. Box 123, Nigeria

**Keywords:** HPV, Africa, Sub-Saharan Africa, girls, women, cervical cancer, vaccination, human papillomavirus, vaccine hesitancy

## Abstract

Background: Cervical cancer, caused by human papillomavirus (HPV) infection, is the second-largest cancer killer of women in low- and middle-income countries. The brunt of the global burden is borne predominantly in Sub-Saharan Africa. In 2020 alone, 70,000 of the 100,000 infected women in Africa died from it, thereby making up 21% of global cervical cancer mortality. The introduction of the HPV vaccine into the National Immunization Program was expected to change the trajectory. However, uptake of the vaccination has been poor, especially for the second dose. Only about half of the countries in Africa currently provide the vaccine. Without urgent intervention, the 2030 global cervical cancer elimination targets will be undermined. The study aims to understand the key challenges facing the HPV vaccine and to develop a roadmap to accelerate the uptake. Method: Fourteen countries were purposively included using a cohort design methodology and the investigation spanned March–July 2023. The Africa region was stratified into three focus-group discussion cohorts (Abidjan, Nairobi and Dar es Salaam), comprising pre-selected countries that have already and those about to introduce the HPV vaccine. In each country, the EPI manager, the NITAG chair or representatives and an HPV-focal researcher were selected participants. The methods involved a collaborative and knowledge-sharing format through regional and country-specific discussions, plenary discussions, and workshop-style group missions. Results: The study reached a total of 78 key stakeholders, comprising 30 participants in cohort one, 21 in cohort two and 27 in cohort three. Key outcomes included the prevalence of declining HPV2 vaccination across all countries in the region; country-specific barriers impeding uptake were identified and strategy for accelerating vaccination demand initiated, e.g., utilizing investments from COVID-19 (e.g., electronic registry and multisector coordination); individual countries developing their respective HPV vaccination recovery and acceleration roadmaps; the identification and inclusion of a zero-dose catch-up strategy into the vaccination roadmaps; support for a transition from multiple-doses to a single-dose HPV vaccine; the incorporation of implementation science research to support the decision-making process such as vaccine choices, doses and understanding behavior. Conclusion: Beyond research, the study shows the significance of scientific approaches that are not limited to understanding problems, but are also solution-oriented, e.g., development of roadmaps to overcome barriers against HPV vaccination uptake.

## 1. Introduction

Human papillomavirus (HPV) is gender-neutral, as both men and women have a 50% risk of being infected at least once in their lifetime [1]. Cervical cancer, caused by HPV infection, is the fourth most frequently diagnosed cancer and the fourth leading cause of cancer-related death among women globally [2,3]. Low-resource countries including those in Sub-Saharan Africa (SSA) have the highest infection burden of the disease with an estimate of 84% and related deaths at 88% [1,4]. In 2020, the number of new global cervical cancer cases stood at over 600,000 with 340,000 deaths, with 90% of these new infections and deaths occurring in low- and middle-income countries (LMIC) [5]. In the same year, in the Africa region alone, 100,000 women were infected, of whom about 70,000 died, making up 21% of global cervical cancer mortality [6]. Therefore, the brunt of the global HPV burden by region is borne predominantly by SSA, at an average of 24% [1,2]. HPV infects basal keratinocytes of the mucosal and cutaneous epithelia, as well as being the common cause of dermatologic diseases, in addition to other variances of cancers including cervical [7]. 

HPV vaccination has been scientifically trusted to be effective in reducing HPV-induced cancers, especially cervical [2]. With the introduction of the HPV vaccine into National Immunization Programs (NIPs), it is expected to change the trajectory of the disease and also the burden, especially in the SSA region. Unfortunately, compared to other regions, SSA has made limited progress in the implementation of the HPV vaccination program [8,9,10]. In countries within SSA where the vaccine has been introduced, coverage for the last dose averages at 20% (95% CI: 5–39%) [11]. Only about half of the countries in Africa currently provide the HPV vaccine through the NIPs [5]. Similarly, data for cervical cancer screening in SSA show only 19% of women attend screening programs, i.e., from as low as 0.7% in Benin to 46% in Namibia [12]. Without urgent intervention, the current HPV vaccination status of SSA will undermine the 2030 cervical cancer-elimination target. 

Since the introduction of the vaccine against HPV and consequently its introduction into NIPs, its uptake has been below expectation, compounded by the COVID-19 pandemic. Furthermore, studies in 13 SSA countries show that decreased access to healthcare facilities is a barrier to HPV vaccination uptake [13]. However, besides access issues, even in countries where the HPV vaccine is available, the uptake has remained abysmally low [8,9,10,14,15]. Of course, the COVID-19 pandemic further added to the challenges. Thus, the already limited healthcare resources were diverted to the pandemic response. Also, due to the COVID-19 lockdown, access to eligible girls dropped and has not returned to the pre-pandemic level [16,17,18]. Similarly, in countries where the HPV vaccination program was to be introduced, misinformation associated with the COVID-19 pandemic, including the COVID-19 vaccine, has hindered such efforts [19,20,21,22], while in others, where it has been introduced, the earlier gains have been eroded [23,24,25,26]. 

The World Health Organization (WHO) Cervical Cancer Elimination Strategy set a target of 90% HPV vaccination coverage (for girls, by the age of 15) to be achieved by 2030 [27,28]. However, as reiterated above, uptake or introduction of the HPV vaccine has been challenging in some African countries, and further negatively impacted by the COVID-19 pandemic. The WHO position paper on HPV vaccines issued in December 2022 gave countries the possibility to reduce the dosing schedule and extend the cohorts to be vaccinated up to the age of 20, so as to remove some of the logistical and financial barriers to HPV vaccination introduction into their NIPs and to improve vaccination coverage rates (VCRs) [29,30]. Understanding the barriers and the key enablers of HPV vaccine coverage and/or introduction is crucial to achieving the 2030 agenda of cervical cancer elimination.

**Figure 1:**
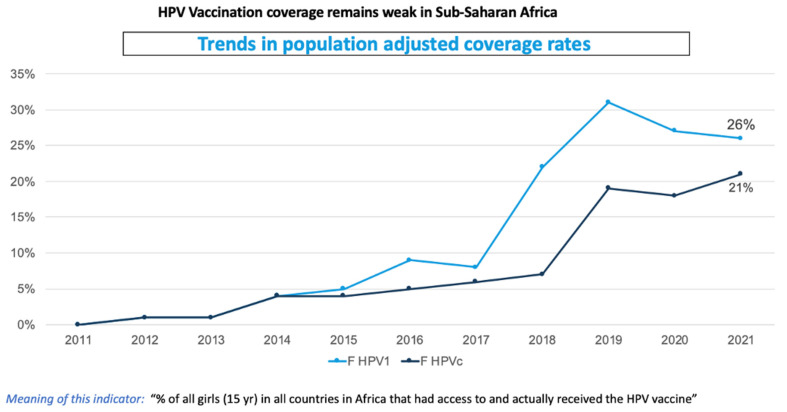
Population-adjusted coverage in Africa (2011–2021) [31].

HPV vaccination uptake in the region has not been as successful as was expected, with the second dose performing even worse in most of the SSA countries, as seen in Figure 1 [31,32,33]. In this context, and taking lessons from previous stand-alone symposia on HPV vaccination held in Africa, stakeholders in collaboration with the WHO Regional Office for Africa, regional partners and the countries’ Ministries of Health have organized this regional knowledge-sharing focus group study on HPV vaccination acceleration and/or introduction. The primary target groups were the countries’ Expanded Program on Immunization (EPI) managers, the National Immunization Technical Advisory Group (NITAG) chairs or representatives and HPV researchers/focal persons, among other state and non-state actors. 

The goal of the study was to understand the country-specific statuses and challenges facing HPV vaccination uptake and/or introduction in the respective SSA countries, and to proffer strategies for maximizing uptake. The primary outcome was to mentor state actors towards developing country-specific post-COVID-19 recovery roadmap for accelerating HPV vaccination and/or its introduction into SSA. The specific objectives include: providing scientific updates to stakeholders on HPV vaccination in SSA; sharing experiences of enablers of the challenges facing HPV vaccination uptake or introduction in the SSA countries; and developing country-specific roadmaps to accelerate HPV vaccination in SSA countries. 

## 2. Methods 

**Figure 2:**
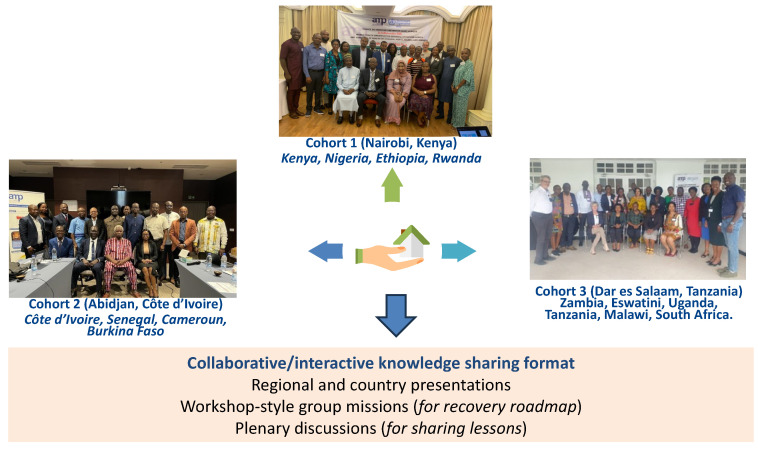
Focus-group discussion cohorts.

Figure 2 highlights the methodological design frame. A cohort-design method was the implementation technique used [34]. The SSA region was stratified into focus-group discussion (FGD) cohorts and pre-selected countries were included based on high and low HPV coverage, and also countries that have just introduced (e.g., Eswatini and Burkina Faso) HPV vaccination into the NIPs and those that are about to do so (e.g., Nigeria). A total of 14 countries were included in the FGDs. The stratification generated three cohorts. Cohort one took place in Nairobi (Kenya) on 23–24 May 2023 and comprised Kenya, Nigeria, Ethiopia and Rwanda. Cohort two met in Abidjan (Côte d’Ivoire) on 30–31 May 2023 and comprised Côte d’Ivoire, Senegal, Cameroon and Burkina Faso. Cohort three took place in Dar es Salaam (Tanzania) on 6–7 July 2023 and comprised Zambia, Eswatini, Uganda, Tanzania, Malawi and South Africa. 

These FGDs adopted a collaborative and interactive knowledge-sharing format using regional and country presentations, plenary discussions, and workshop-style group missions. The regional overview was guided by facilitators from the WHO Regional Office for Africa, the Solina Centre for International Development and Research, the Dose-Reduction Immunobridging and Safety Study (DoRIS), Jhpiego, Merck Sharpe & Dohme (MSD) and Agence de Médicine Préventive (AMP) Afrique, among others. The country-specific overviews (strengths, weaknesses, opportunities and threats (SWOT) analysis) were presented by the EPI managers for each country, where the HPV vaccination status and challenges faced were reported. Participants were mentored in workshop-style sessions to develop a country-specific post-COVID-19 recovery roadmap using pre-defined tools. 

The FGDs focused on the importance of HPV vaccination in preventing cervical cancer, given that 75–90% of sexually active women will be exposed to HPV infection during their lifetime and, even more, and more so that vaccination is essential to reduce persistent diseases and associated cancers [35]. The goal of eliminating cervical cancer was reiterated, with an incidence threshold set at less than 4 cases per 100,000 women [36].

An integrated part of the focus group was the workshops designed to help each country develop a recovery plan or an HPV vaccination acceleration or introduction plan, which would be implementable upon return home. The workshop outcomes, which were presented by each country, involved a SWOT analysis of the HPV vaccination program or introduction plans of each country, defining the strategic vision/goal and then mapping into categories the priority strategies envisaged for achieving set goals, such as health policy, demand generation, surveillance, implementation research and vaccine logistics. The priority strategies were backed-up with actionable activities, timelines, and expected outcomes/impacts. The finalized roadmaps were presented at plenary of each cohort for feedback and input from other countries. 

Key eligibility criteria for inclusion in the Africa HPV vaccination focus-group discussion cohorts: at least two countries from each regional bloc of SSA (East, West, Central and Southern Africa); a mix of countries with high and low HPV coverage (i.e., those below and above 50%); the EPI manager for the selected countries; the chair or member of NITAG for the selected countries; an HPV researcher/country focal person for the selected countries; representatives from the WHO Regional Office for Africa and country offices of selected countries; and representatives of strategic development partners in the selected countries. 

The data were analyzed using a thematic and cohort-based approach, which sought to summarize the data in a cohort or stepwise fashion and assess the themes based on topics. Convergence and divergence of views in each of the cohorts were identified after analyzing each individual submission and categorized them based on the aggregation of the cohort subject outcomes. The participants were coded based on the numbering of the cohorts and an identifier was given (e.g., cohort one as C1_01–30, cohort two as C2_01–21 and cohort three as C3_01–27). The transcribed data were coded and structured along the study objectives, cohort format and themes explored. The coding and themes development were inductively generated. 

## 3. Results 

The focus group discussions reached a total of 78 participants (key stakeholders), comprising 30 participants in cohort one (Nairobi, Kenya), 21 in cohort two (Abidjan, Cote d’Ivoire) and 27 in cohort three (Dar es Salaam, Tanzania). The focus groups revealed significant disparities in vaccination coverage as well as differences in the contexts of the implementation of HPV vaccination in the SSA region. 

### 3.1. Key Outcomes: Cohort One 

The cohort one stakeholders delved into a series of themes including cervical cancer burden and the introduction of the HPV vaccine in Sub-Saharan Africa. The cohort expanded on the new Strategic Advisory Group of Experts on Immunization (SAGE) recommendation (“WHO Position and SAGE Recommendation for HPV Vaccination”), stressing that HPV vaccines should be introduced early in a coordinated manner and highlighted that the single-dose HPV vaccine offers substantial protection comparable to multiple doses [29,30,37]. Table 1 highlighted the key outcomes. 

### 3.2. Key Outcomes: Cohort Two 

The experiences and initiatives of the four pioneering francophone countries (Senegal, Cameroon, Burkina Faso and Côte d’Ivoire) during this study offered invaluable insights (see Table 2), in addition to serving as a roadmap to guide other francophone African countries in the planning and successful implementation of HPV vaccination programs.

Senegal holds the distinction of being the first Gavi-supported francophone African and West African country to introduce the HPV vaccine into her NIP [38]. However, by 2022, the coverage stood at just about 22%, and the COVID-19 pandemic contributed partly to this [39]. Her aim to eliminate cervical cancer as a public health crisis by 2035 seems a bit shaky unless drastic actions are taken. In Côte d’Ivoire, HPV vaccination coverage has stagnated between 34% and 41% since its introduction in 2020 [40]. The low rates are due to vaccine hesitancy among others: insufficient communication, low engagement with HPV vaccination by certain health workers, poor communication on cancers in general, and lack of micro-planning for routine HPV activities [2,41]. Cameroon introduced the HPV vaccine in 2020, alongside COVID-19 vaccination, but faced mass refusal, particularly among religious leaders, leading to a mere 5% coverage rate in 2021 [42,43]. Other challenges identified included gaps in communication, weak leadership commitment, and insufficient partner mobilization [42,43]. In 2022, Burkina Faso launched the HPV vaccination in response to growing demand, with a twofold plan ensuring 95% of 9-year-old girls and boys receiving an HPV vaccine dose by the end of 2024 and achieving at least 95% vaccination among girls aged 10–18 during a catch-up campaign [44]. 

### 3.3. Key Outcomes: Cohort Three 

The cohort collectively brainstormed on some fundamental pull and push factors driving low HPV vaccine uptake in the region and how to address them (see Table 3). They included the inadequate resources needed to manage cervical cancer in SSA, such as human resources (specialists); infrastructure (vaccine, screening services and treatment), finance (insufficient domestic and donor funding) and cancer registry (limited availability of population-based cancer registries); prevalence of late presentation leading to delayed treatment and then poor health outcome, which are associated with limited awareness and knowledge combined with fear and stigmatization. In addition, there are weak monitoring and evaluation (M&E) systems in the NIP structures; and poor adherence to guidelines, among others. 

Malawi introduced the HPV vaccine into the NIP in 2019 in a single-age cohort of 9-year-old girls; however, since the coverage peaked at 83% in 2019, it has rapidly declined to 14% for HPV1 and 12% for HPV2 [45,46]. For Tanzania, following a successful pilot program, Tanzania scaled-up and introduced the HPV vaccine into the NIP in April 2018 for 14-year-old girls, using the routine immunization delivery strategy, and not via campaigns or other point-in-time delivery strategies. In Zambia, the HPV vaccine introduction was in June 2019, and vaccination coverage since then has dropped significantly from 75% in 2019 to 39% in 2021 [47]. The stakeholders attributed the decline essentially to vaccine hesitancy and misinformation. Uganda was among the first countries in SSA to introduce HPV vaccine targeting 10-year-old girls. However, the burden of cervical cancer is still high, with new cases at 6,959, and 4,607 deaths; and with 57% HIV prevalence (adults 15–49 years) [48]. Although the intervention approach was both school-based and community outreach, and stakeholders cited “early engagement of teachers as partners was helpful”–(C3_03, C3_13, C3_19, C3_21), nevertheless, Uganda still struggles with problems of rumors and misconceptions about the HPV vaccine. In South Africa, the high level of commitment and the multi-sectoral involvement of government ministries and civil society organizations played important roles in the successful introduction and implementation of the program. However, the coverage for the first dose has been consistently declining from above 80% in 2014 to 3% in 2022, although affected partly by the COVID-19 pandemic [11,49]. Also, the HPV vaccination point of entry has not been able to move beyond the public schools in South Africa. Meanwhile, Eswatini, who recently launched an HPV vaccination program during the period of this study (June 2023), had a lot to share including resource mobilization and the planning processes. The Manzini district vaccinated the highest number of girls across all ages out of the 9–14 cohort targeted, while 11-year-old girls received the highest number of doses of the first shot in the country. 

### 3.4. The Single-Dose Efficacy 

**Figure 3:**
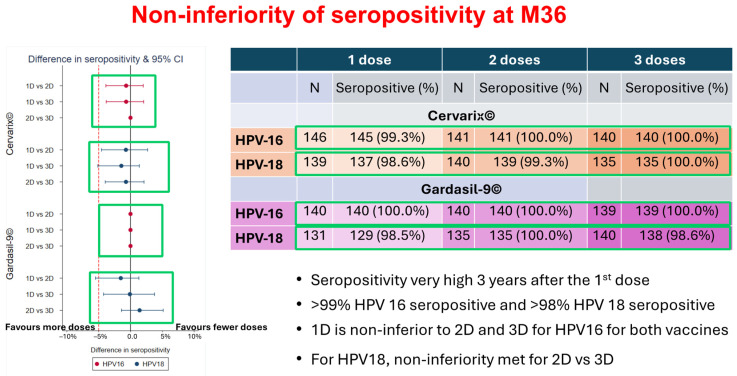
DoRIS study efficacy of single-dose versus multiple doses [29].

The study on Dose-Reduction Immunobridging and Safety (DoRIS) as presented in the focus group roundtable by DoRIS showed that the single-dose HPV vaccine could reduce cost and simplify delivery; increase the accessibility and sustainability of HPV vaccination programs especially for LIMCs; increase acceptability by girls; high seropositivity (>98%) for HPV16/18 at M36 with one dose of Cervarix^®^ and Gardasil9^®^; stabilize antibody levels and trajectories over time from M12 and consistent across studies; and avidity [29]. The DoRIS study showed that there is no difference in terms of efficacy between one, two, or three doses of the HPV vaccine as seen Figure 3. For the targeted ages of 9–14-year-old girls, the one dose (1D) immune response in DoRIS was non-inferior compared with three different studies where 1D efficacy was observed. Therefore, the DoRIS study alongside the majority of participants supported the single-dose recommendation in this age group in SSA. 

### 3.5. Development of Country-Specific Roadmaps

**Figure 4:**
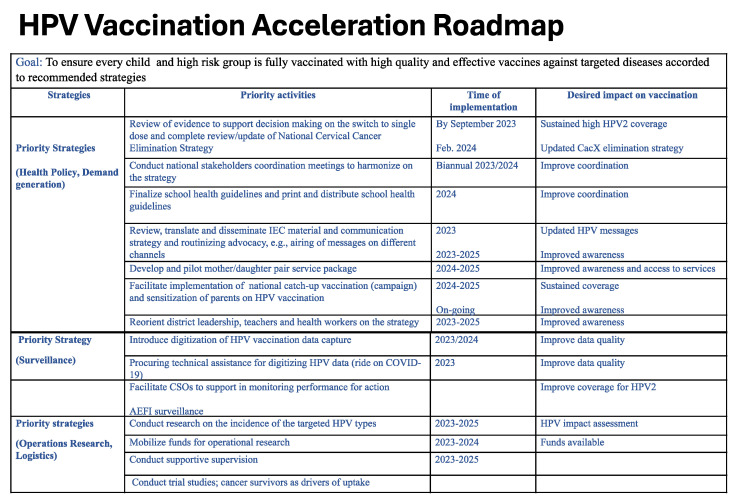
HPV Vaccination acceleration roadmap template.

Each cohort’s FGD ended up with a workshop, where each country developed a roadmap after the SWOT analysis of their current HPV vaccination program. The relationships that have been established through this study and between the participating countries were crucial to achieving the common goals of increasing HPV vaccination post-COVID-19 and thus reducing mortality and morbidity associated with cervical cancer in SSA. Figure 4 displays a summary template or sample of the developed country’s roadmap.

## 4. Discussion

The goal of the study was to understand the barriers facing HPV-vaccine demand and to leverage stakeholder-led strategies to maximize uptake in SSA. Beyond the impressive participation by the 78 most important stakeholders in the region across 14 countries, the key outcomes included the following: the prevalence of declining HPV2 vaccination across all countries in the region; country-specific barriers impeding uptake were identified and a strategy for accelerating vaccination demand was advanced, e.g., utilizing investments from COVID-19 (e.g., electronic registry and multisector coordination); individual countries developed their respective HPV vaccination recovery and acceleration roadmaps; the identification and inclusion of a zero-dose catch-up strategy into the vaccination roadmap; support for a transition from multiple-to-single-dose HPV vaccine; incorporation of implementation research to support the decision-making process for vaccine choices, doses and understanding behavior.

As part of strengthening communication and demand-generation activities, it is important to focus on information and community awareness to improve knowledge around the HPV vaccine using negative framing, by regularly linking it to a consequence, which is cervical cancer. Constant community mobilization is important for improving vaccination coverage and countering misinformation. Seizing all awareness opportunities that a country’s EPIs can find is important. Among the 14 countries, Tanzania, Malawi and Uganda need to do more in this area.

The M&E activities to enhance program-performance tracking need adjustments across all the programs in the region. The study showed weak M&E systems in the EPI or NIPs of countries. A supportive M&E system should be integrated properly into the EPI program plans and decentralized to sub-national levels as well. To this end, it is critical that a budgetary cap of a certain percentage be placed on a country’s HPV program, specifically dedicated to M&E.

Promoting the transition to a single-dose vaccination will help countries in SSA overcome the burden of costs and help to simplify vaccine delivery, especially in the post-Gavi-support era. Multidose vaccine schedules are expensive and complex to deliver, and the logistics associated with the vast and scattered population peculiar to the SSA region make it less likely that the region will achieve the elimination target of 2030 unless a new approach (e.g., single-dose strategy) is explored. While this is becoming very popular among countries especially after the WHO recommendation, some others such as Ethiopia, Rwanda, Uganda are still not there, hence a more evidence-based study on single-dose efficacy is required. NITAGs have a lot of work to do here in terms of recommendations to their respective countries EPI.

The expansion of education and HPV vaccination to a broader target population including boys will have a positive impact on the girls as well. Also, it would demystify the conspiracy theories and the talking-points of anti-vaxers around the suspicion of the HPV vaccine, especially being a vaccine for “girls only” in the region [50,51]. Similarly, a critical component of the priority strategies should include the mapping of vaccine-hesitant groups in order to provide tailored messaging to this unique demographic or population. Hesitant groups need to be identified using population-based research and a tailored intervention implemented, primarily with a unique messaging strategy.

Continuous implementation research either as stand-alone or part of the M&E framework of a country’s EPI is essential. It will provide data on vaccine effectiveness and the changing or consistent nature of community behavior, all aimed at using evidence to improve the HPV vaccination program at national and sub-national levels. Also, it will be important that all countries strengthen their HPV vaccination plan after the peak of the COVID-19 pandemic, especially for those with high numbers of zero-dose and under-vaccinated girls (e.g., Tanzania and others).

Most of the HPV vaccination interventions are school-based, hence not comprehensive enough to cover out-of-school girls, especially in hard-to-reach communities. The creation of outreach vaccination campaigns for out-of-school-girls using mobile teams and mobile campaigns similar to the polio strategy would be effective in the region. Similarly, the inclusion of the HPV vaccine in the School Health Guidelines and School Health Policy could help normalize the familiarization of adolescent boys and girls with knowledge of HPV and its prevention using a vaccine. There is a need for synergy between the Ministries of Health and Education in the SSA countries.

In francophone Africa, the introduction of the HPV vaccine is progressing at a slow pace, with coverage significantly lagging behind other regions. Among the 54 African countries, only four francophone nations—Burkina Faso, Côte d’Ivoire, Cameroon, and Senegal—have adopted the vaccine. This situation underscores a considerable disparity between Anglo- and Francophone African countries within the region. The slower pace of adoption may mirror underlying challenges tied to communication, cultural acceptance, infrastructure, political commitment, and resource allocation. To accelerate the pace and broaden the vaccine coverage in the Francophone African region, it might be essential to collaborate efforts, develop tailored strategies, and leverage shared learnings from countries that have successfully introduced the vaccine.

The focus groups created a vital platform for the comprehension of the severe impact of cervical cancer and helped lay specific emphasis on the SSA countries, thereby facilitating vibrant and interactive discussions centered on cervical cancer epidemiology, country-specific elimination plans, and the crucial role of HPV vaccination in preventing cervical cancer. The discussions revisited the global goal of eradicating cervical cancer, delineating an ambitious yet attainable target of fewer than four cases per 100,000 women. This target effectively framed the discussion, steering it towards tangible actions and measurable progress.

### Strategic Discussions for Improving HPV Vaccine Uptake in SSA

The focus-group cohorts were a big trove of data for experience-sharing and lesson-drawing between the countries to scale up HPV vaccination or introduction into their NIPs. The focus-group cohorts helped the countries to appraise their status through SWOT analysis and then understand the barriers, and the enabling and mitigation strategies needed to overcome the current and anticipated challenges. Other implications of the study outcomes include:

- One of the most significant outcomes of the focus-group cohorts was the shared collaboration of the countries to develop post-COVID-19 HPV vaccination recovery roadmaps, with specific targets to overcome barriers impeding uptake or introduction.
- The resolve amongst the countries and partners to use the roadmap developed during the focus-group cohort discussions as an updated national strategy for accelerating uptake or introduction.
- The focus groups helped to unravel the problems of vaccine coverage and demand disparities across the Francophone Africa region, in countries such as Senegal, Cameroon, Burkina Faso and Côte d’Ivoire. Also, a challenge to expand HPV vaccine launch to other Francophone African countries, or at least across the remaining seven Francophone West African countries.
- The study discovered a lack of implementation research within all the countries’ HPV vaccination programs, in addition to limited research on HPV circulating serotypes and assessment of the clinical, histological and immunological efficacy of a single-dose.
- The study brought to the fore the prevalence of the declining HPV2 dose vaccination across countries in the region, thereby necessitating brainstorming on how to address it, e.g., using new knowledge acquired from the cohorts and lessons from peers that are doing well, such as Rwanda and Ethiopia. Also, the idea of using single-dose as a remedy to the negative trend.
- The use of wives of political figures such as first ladies and female elected leaders as the faces of the HPV vaccination campaign seems a positive strategy. The study outcome showed that countries that adopted this strategy had better uptake than others that did not.
- It was insightful to understand that critical barriers or challenges to HPV vaccination uptake common to all the countries, besides Rwanda, were budget constraints (funding), data quality issues (DHIS 2 versus administrative data) and misconceptions about HPV vaccine or vaccine hesitancy. Myths and misconceptions around the HPV vaccine seem to be recurrent impediments to HPV vaccination uptake among the 14 countries. These were primary ingredients for inflaming vaccine hesitancy.
- Any national HPV vaccination strategy using a school-based approach must integrate the school calendar into its implementation plans, otherwise it will be ineffective, i.e., full involvement from the Ministries of Education in HPV vaccination produced better coverage, based on coverage data from countries who had done so compared to others in the cohorts that had not.
- Across the countries, the outcomes showed there was a problem of inaccurate data reporting which led to disparities between administrative and DHIS 2 data. This data problem needs to be reconciled and systematically aligned across the SSA region for effective decision-making. The COVID-19 pandemic data system could be a useful resource that could be exploited.
- The study approach and methods were very novel, because besides being an empirical study, they facilitated peer mentorship and learning among African countries, especially sharing lessons on strategies that have worked and those that have not. This was inspirational for countries who have just introduced HPV vaccination or are about to do so, e.g., Eswatini, Burkina Faso, Cameroun and Nigeria.
- The study discussion led to clarification of the discordance in the dosage regimen adopted by various countries, from three doses to two doses and a single-dose. There was unanimity across the cohorts that all countries should make a decision on the dosage based on the peculiarities of their own country’s situation. While some countries such as Nigeria, Kenya, Cameroon are ready for the single dose, others such as Ethiopia, Rwanda, Uganda are not there yet. However, the feelings of the majority of the participants were that the single dose is much better for the particular situation in Africa.
- The poor or stagnant coverage in Côte d’Ivoire and some other countries is largely attributable to vaccine hesitancy and further complicated by other factors such as ineffective communication, low appropriation of vaccination by health workers, poor micro-planning and inadequate awareness. Although Senegal’s experience of a drastic decline in coverage from 94% in 2019 to 22% in 2022 was attributed primarily to the uncurbed impacts of misinformation and inadequate community engagement, both situations must be considered independent of each other, despite both being in the Francophone African cluster.
- Broad acknowledgement of Senegal and Cameroon’s struggles which were found to be particularly linked to opposition from religious leaders, weak implementation strategies and a lack of commitment in some districts due to poor engagement was significant. The roadmap developed during the study has incorporated these inputs.
- Strategies to optimize coverage were uniquely maximized in Rwanda, Ethiopia, Nigeria, Kenya and a few other countries by leveraging a blend of school-based strategies, community outreach, and health facility delivery. The blend was particularly aimed at reaching in-school and out-of-school girls. This provides template for replication in the region.
- A significant outcome of the study demonstrated that, despite consensus on common strategies for improving HPV vaccination uptake, the success of each country’s approach is intricately linked to maximization of their unique local circumstances, opportunities, and how they are able to mitigate obstacles.
- Unique challenges were highlighted for some countries: local political unrest and unreliable administrative data (Ethiopia); sustainability of financing existing school-based initiatives (Kenya); limited experience with adolescent vaccination (Nigeria); the polarity in a country’s capacity to reach ambitious targets (Burkina Faso); inadequate HPV vaccine information and communication strategies (Côte d’Ivoire); lack of support from political and religious leaders (Cameroon); insufficient community mobilization to dispel rumors and misinformation (Senegal); discordance between parents and school authorities (Eswatini); poor multi-sectoral collaboration between the Ministries of Education and Health (Malawi and Tanzania); inadequate human resources to deliver on school outreach needs (Uganda); supply chain issues (Zambia); high turnover of HCWs (South Africa), among others.

The limitation of this study was the inability to include more SSA countries in the sample. However, based on the cohort design and the eligibility, 14 countries and the representation of the most important or key actors in each country’s immunization program were adequate for any scientific conclusion. Second, the tendency of FGD participants to free-ride (i.e., give socially desirable answers) in group settings is common compared to individual interviews, which are better at mediating such effect. However, it is an effective method for this particular study, because it allows participants who share similar or different experiences to feed off each other’s responses, and this generates new ideas which might not have been raised in an individual interview.

## 5. Conclusions

**Figure 5:**
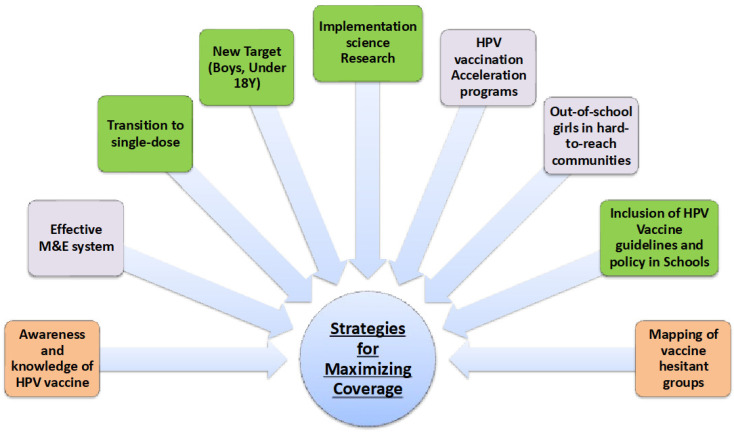
Strategies to foster HPV vaccine uptake.

The study was fully optimized in many respects, principally because participants from 14 countries shared ideas and opinions on the mutual challenges associated with HPV vaccination uptake, the reasons for such outcomes and the approaches suggested to address them. The primary outcome was achieved, because the country-specific challenges driving low HPV vaccination uptake were reported and recovery roadmaps for improving HPV vaccine demand were developed on a country-by-country basis. The participants, most of whom are the coordinators of EPI in their respective countries and senior government representatives, were nudged to adapt the study outcomes (e.g., the roadmaps) and work together to achieve the common goal of reducing the burden of cervical cancer in the SSA region. It is essential that there is continued support for these countries in their efforts to overcome the challenges identified and ensure equitable access to HPV vaccination, in addition to fighting against the misconceptions and misinformation that breed vaccine hesitancy. Collaboration between SSA countries, partners, public health actors and the scientific community is crucial if the region’s cervical cancer burden is to be reduced and for collectively working on the identified themes below (as shown in Figure 5) to achieve HPV vaccine uptake.

Vaccine coverage and demand disparities across SSA, especially the Francophone African region underscores the need to strengthen communication, awareness and community engagement efforts in each of the four countries and at least expand the launch of HPV vaccination to the other Francophone African countries. The study brought to bear the significance of scientific approaches that are forward-looking and not just about understanding problems, but are also solution-oriented, one of which was the development of roadmaps with specific targets to overcome country-specific barriers to HPV vaccination uptake. Finally, HPV multi-dose vaccine schedules are expensive and complex to deliver in low-resource settings. Therefore, the SSA countries must evaluate the single-dose HPV vaccine as the best possible option for the obvious reasons discussed in this study.

## 6. Recommendations

Based on the outcomes of the study, the following recommendations are proffered:

1. A dual anchor system should be initiated to support countries to overcome not only the decline in HPV2 uptake, which the sharing of lessons learnt among peers could help to mitigate, but also to overcome their population’s perception issues that contribute to low uptake or vaccine hesitancy.
2. The first anchor should focus on implementation research to support the EPI program with scientific evidence to build resilience and steady uptake. This recommendation is evidence-based owing to a systematic review finding: “To achieve the WHO target by 2030, we call for studies to understand the barriers and facilitators from the perspectives of stakeholders in order to support the decision-making processes and information required to implement recovery strategies in LMICs” [3].
3. The second anchor, based on the two positive outcomes of this study (sharing opinions/experiences and developing recovery roadmaps), suggest future public health research in low resource settings, such as SSA, should be multifaceted, i.e., understanding the drivers of problems and also finding approaches for solutions.
4. Several countries attributed the decline of HPV vaccination to increased rumors and misinformation as well as to disinformation campaigns. So, concerted efforts using the traditional institutions (religious and cultural) are advised, in addition to the retraining of healthcare workers.
5. NITAGs of various African countries should consider the recommendations for the single-dose option. Multi-dose vaccine schedules are expensive and complex to deliver, and the logistics associated with vast and scattered populations make it less likely that the region will achieve the 2030 target.
6. Strengthening coordination and collaboration. One of the obvious outcomes was the need for close coordination and collaboration because of the similar peculiarities of the challenges surrounding HPV vaccination in the region. There is a need to:
  - Strengthen the intersectoral coordination around HPV vaccination, involving key actors such as the education system, professional associations and community leaders.
  - Promote exchanges and cooperation between SSA countries by creating a research network for sharing information and scientific knowledge.
  - Organize inter-country roadmap appraisal in the form of a bi-annual experience-sharing focus group on HPV vaccination.
  - Revitalize initiatives on the association of first ladies with HPV vaccination. Lessons from peers on how to do so abound.
7. Besides launching a dedicated program of implementation research for HPV vaccination to support the HPV programs, there should also be a strengthening of the diagnosis and monitoring of circulating HPV genotypes in the African region and more inter-country studies to assess the clinical, histological and immunological efficacy of the single-dose HPV vaccine.

## Figures and Tables

**Table 1:** Key Strategies for Maximizing Coverage.

“There is need for school-based strategies, community outreach, and health facility delivery to optimize coverage and reach out-of-school girls”–C1_01-. “We must integrate HPV vaccination with other health services (e.g., Rwanda, Ethiopia, Kenya and Nigeria)”–C1_02. ○ “…Ethiopia: Merge HPV vaccination with COVID-19 and Routine Immunization (RI)”–C1_04. ○ “…Rwanda: Full integration of HPV vaccination into routine immunization and cancer prevention”–C1_02. ○ “…Kenya: Strengthened linkages between health facilities and schools”–C1_05. ○ “…Nigeria: Drawing from prior experiences with new vaccine introductions. In addition, integrating critical outcomes of this discussions into the 2023–2027 National Cancer Control Plan, because 7,968 annual cervical cancer deaths in Nigeria is alarming”–C1_03. “…the need for strong governmental commitment. (e.g., Rwanda with over 90% vaccination coverage)”–C1_25. “…focus improvement on facility-based HPV vaccination delivery (e.g., Kenya, achieving 75% for HPV1 and 40% for HPV2)”–C1_09. “…essentials of rigorous data monitoring (e.g., Ethiopia)”–C1_29. “Emphasizing single-dose recommendation for cost and logistical efficiency. Although, there is need for more scientific evidence supporting the single dose’s benefits for individual country decisions. While Ethiopia is still considering a single-dose option, Nigeria and others has chosen this route after advice from their NITAGs”–C1_01. “Every country recognized the importance of collaboration with various stakeholders, including educational authorities in Ethiopia, religious and tribal leaders in Kenya, and governmental figures in Rwanda and so on”–C1_30. “…the importance of research on vaccine’s immune-response and efficacy to boost and sustain uptake in high-risk populations”–C1_06. “The four nations shared core principles in their HPV vaccination initiatives, such as community engagement, school-based methods, and confronting challenges like funding constraints and misinformation. Yet, they exhibited differences in performance metrics, precise strategies, integration with other health initiatives, and future planning”–C1_23. “Rwanda’s triumphant efforts provide valuable insights, while Nigeria’s introduction added a novel dimension. The discussions here underline that common strategies exist, but the success of each country’s approach is intricately linked to its unique circumstances, opportunities, and obstacles”–C1_10.
Challenges and Common Hurdles: “…Ethiopia: The country’s contention with local political unrest and shaky administrative data”–C1_13. “…Kenya: Struggling with sustainable financing for school-based initiatives”–C1_23. “…Nigeria: Anticipating challenges during the introduction phase and learning from regional peers”–C1_03. “…Rwanda: Reported fewer challenges, indicative of a well-grounded program”–C1_02. “Common to all (except Rwanda): “Budget constraints, data quality, and misconceptions leading to vaccine hesitancy”–C1_01.

**Table 2:** Key Strategies for Maximizing Coverage.

“Implementation of field-based research on HPV circulating serotypes is essential”–C2_05 “Assessment of the clinical, histological and immunological efficacy of single-dose HPV vaccination is still insufficient for strong country-level decision-making on the matter”–C2_21. ○ “Extension to new target population such as boys (Burkina Faso, Cameroon) and girls aged 9 to 18 years old (Burkina Faso) could impact general HPV vaccine uptake”–C2_15, C2_19, C2_01, C2_20. “Implementation of the single dose vaccination as recommended by WHO (e.g., Burkina Faso, Cameroon, Côte d’Ivoire)”–C2_02, C2_19, C2_03. “Improvement of NIP micro-planning (e.g., Côte d’Ivoire)”–C2_03. “Strengthen communication activities involving community leaders and mobilizing local partners”–C2_20. “Integration of HPV vaccine into the NIPs (e.g., Cameroon)”–C2_19. “Vaccine coverage and demand disparities across Francophone Africa region underscore the need to strengthen communication, awareness and community engagement efforts in each country”–C2_21. “The focus group discussions allowed key stakeholders to engage on HPV vaccination performance, key challenges and opportunities, thereby opening the need for tailored country specific strategies”–C2_01.
Unique Challenges and Common Hurdles: “…Burkina Faso: Striving to meet ambitious targets, i.e., her target is unrealistic”–C2_02. “…Côte d’Ivoire: Insufficient communication, poor awareness, and vaccine hesitancy–C2_03. “…Cameroon: Strong opposition from religious leaders, communication gaps, and weak leadership commitment”–C2_19. “…Senegal: Rumors, misinformation, and insufficient community activities leads to decline in demand”–C2_11. Common to all: “Concerns about lack of implementation science research on HPV circulating serotypes and assessing the clinical, histological and immunological efficacy of single-dose HPV vaccination”–C2_21.

**Table 3:** Key Strategies for Maximizing Coverage.

“Increasing awareness and knowledge of HPV vaccine (e.g., Tanzania, Zambia, Malawi and Uganda)”–C3_03; C3_07, C3_19, C3_24. “…Effective monitoring and evaluation systems in the NIPs (e.g., Uganda, South Africa)”–C3_23; C3_27. “…Promote transition to single-dose vaccination to help overcome costs and simplify delivery (e.g., Malawi, Eswatini, Tanzania)”–C3_17; C3_30, C3_01. “…Legislative advocacy (mandate) of HPV vaccine for 9–14 years old girls (e.g., Malawi)”–C3_19. “Relaunching of HPV vaccination campaigns (e.g., in Malawi where coverage declined to 14% for HPV1 and 7% for HPV2 in 2022. Also, for South Africa)”–C3_18; C3_28. “Strengthen of HPV vaccination coverage improvement plan (2023–2025) for districts with the highest number of zero dose and under-vaccinated girls (e.g., Tanzania)”–C3_01. “Inclusion of implementation science research in NIPs to understand behavioral and contextual factors driving declining HPV vaccination (especially HPV2…e.g., in Malawi, Tanzania, South Africa, Zambia)”–C3_18; C3_02, C3_28, C3_05. “…incorporation of teachers as HPV vaccination program partners (e.g., Uganda)”–C3_23; C3_01, C3_30. “Strengthening of social mobilization and engagements activities targeting parents (e.g., Uganda, Zambia, Eswatini)”–C3_24; C3_05, C3_29. “Strengthen coordination with the Department of Basic Education to incorporate private schools, mitigate turnover of HCWs and improving social mobilization activities (e.g., South Africa, Eswatini)”–C3_28; C3_30. “…Increasing mobile services for out-of-school eligible girls in hard-to-reach communities (e.g., Eswatini, Uganda)–C3_30; C3_23. “…Identify and use of HPV champions (e.g., Zambia)”–C3_04. “Transition to digitalization of data collection and reporting (e.g., Eswatini, Zambia)”–C3_29; C3_05. “Inclusion of HPV Vaccine in School Health Guidelines and School Health Policy (e.g., Tanzania)”–C3_02. “Mapping of vaccine hesitant groups and providing tailored messaging (e.g., Zambia and others)”–C3_07; C3_24. Common to all: “Multi-sectoral collaboration, especially between ministries of health and education is essential”–C3_01.
Unique Challenges and Common Hurdles: “…Uganda: Prevalence (57%) of HIV among target group and inadequate human and material capacities to meet needs of school outreaches”–C3_25. “…South Africa: Inadequate capacity to provide HPV vaccination beyond public schools and high HCWs turnover”–C3_26; C3_27. “…Eswatini: Significant communication gap between parents and school authorities”–C3_30. “…Malawi: Rumors and misconception of HPV vaccine as COVID-19 vaccine”–C3_18. “…Tanzania: Exclusion of out-of-school girls due to inadequate outreach activities”–C3_03. “…Zambia: Frequent school change among eligible girls thereby distorting HVP2 and supply chain issues”–C3_07. Common to all: “Poor collaboration between ministries of education and health, global HPV vaccine glitch/shortage, data quality issues”–C3_30.

## Data Availability

The datasets used and/or analyzed during the current study are available from the corresponding author on reasonable request.

## Abbreviations

EPI	Expanded Program on Immunization
HPV	Human papillomavirus
NITAG	National Immunization Technical Advisory Group
SAGE	The Strategic Advisory Group of Experts (SAGE) on Immunization
SSA	Sub-Saharan Africa
CBO	Community-based organizations
MSD	Merck Sharp & Dohme
MCH	Maternal and care health
KENSHE	Kenya Single HPV Vaccine Study
PLWHIV	People living with HIV/AIDS
NCD	Non-communicable diseases
WHO	World Health Organization
MOH	Ministry of Health
DoRIS	A Dose-Reduction Immunobridging and Safety Study
NIP	National Immunization Program
CSOs	Civil Society Organizations
NVI	New vaccines introduction
NPHCDA	National Primary Health Care Development Agency
Q&A	Questions and answers
HF	Health facilities
RI	Routine Immunization
LMIC	Low- and middle-income countries
HIC	High-income countries
SWOT	Strength, weakness, opportunity and threat
HPV1	First dose of HPV vaccine
HVP2	Second dose of HPV vaccine
M&E	Monitoring and evaluation
AUCDC	African Union Centre for Disease Control
